# Lactic Acid in Diphtheria

**Published:** 1883-10

**Authors:** J. P. Lytle


### ARTICLE VII.

### LACTIC ACID IN DIPHTHERIA.—BY J. P. LYTLE, M.D.

Several years ago I saw a suggestion in one of the medical journals that a spray of dilute lactic acid would dissolve pseudomembrane of croup.

I concluded that if it would do so, it would also dissolve the membranous deposit of diphtheria. Since then I have used it in a number of cases with the happiest results. Where the patient is old enough to permit a thorough spraying of the throat I have not failed to remove the deposit with two or three applications, sometimes with one.

May 4, 1883, 4 P.M., was called to see young B., aged sixteen. Face flushed; pulse quick; temperature 102; throat very painful, and deglutition very difficult. Upon examining his throat I discovered a gray, gelatinous membrane fully one-eighth of an inch thick, covering the entire tonsil, and extending over the soft palate of left side; right inflamed and swollen, but no deposits upon it. I sprayed the throat with the following solution: Acid lactic concent., gtts xxx, aqua ℥i, and had the satisfaction of seeing the membrane entirely disappear. I put him on general treatment, stating that I would call in the morning. I then found the left tonsil and palate clear, but the right tonsil and palate had upon it a deposit fully as extensive as the left had upon it the night before. I again used the spray, and had the satisfaction of seeing the throat clear of deposit when I was through. I continued the general treatment, and left a solution acid lactic gtts x, aqua ℥i, to be used as a gargle. He made a rapid recovery, and there was no more deposit of membrane. To those of your readers who have not used the acid in this way they will find it worthy of trial. I use a double-bulb rubber atomizer with rubber tip.

---

### RESECTION AND EXPORTATION OF THE STERNUM.

The resection of the sternum so far has been executed in Italy by Rizzoli, Mazzoni and Pecchioli. Heidelfer has reported 25 cases of resection of small portions of the bone. Parona reports in the *Independente* a case of a woman fifty-six years old, affected for three years with necrosis of the sternum. An operation was performed with a complete success, as the bone was reproduced almost completely, although the woman was of such an age, and in a debilitated condition, and moreover, there existed a gangrenous condition of the edge where the periosteum was attached. The fact of the bony reproduction in this case is of an exceptional importance, as no one of those operated upon for resection of an extensive portion of the sternum was of such an advanced age as the patient of the author.—*Lo Sperimentale.*

### AXILLARY HYPERIDROSIS OF NAKED PERSONS.

M. Aubert, in a paper read before the Medical Society of Lyons, asserted that under the influence of nakedness the axillæ become moister, and can be seen to change in redder color. M. Aubert believes that this phenomenon produces an elevation of temperature of the arm-pit caused by a general lowering of the temperature of the surface of the body. He, therefore, combats the habit of taking the degree of temperature with the axillary thermometer.—*Lyons Medical.*

